# Enhanced Thermal Conductivity of Polyimide Composites with Boron Nitride Nanosheets

**DOI:** 10.1038/s41598-018-19945-3

**Published:** 2018-01-24

**Authors:** Ting Wang, Mengjie Wang, Li Fu, Zehui Duan, Yapeng Chen, Xiao Hou, Yuming Wu, Shuangyi Li, Liangchao Guo, Ruiyang Kang, Nan Jiang, Jinhong Yu

**Affiliations:** 1 Key Laboratory of Marine Materials and Related Technologies, Zhejiang Key Laboratory of Marine Materials and Protective Technologies, Ningbo Institute of Materials Technology and Engineering, Chinese Academy of Sciences, Ningbo, 315201 China; 2 College of Materials and Environmental Engineering, Hangzhou Dianzi University, Hangzhou, 310018 China; 3 Chemical Engineering and Biotechnology, National Taipei University of Technology, Taipei, 10608 China

## Abstract

A strategy was reported to prepare boron nitride nanosheets (BNNSs) by a molten hydroxide assisted liquid exfoliation from hexagonal boron nitride (h-BN) powder. BNNSs with an average thickness of 3 nm were obtained by a facile, low-cost, and scalable exfoliation method. Highly thermally conductive polyimide (PI) composite films with BNNSs filler were prepared by solution-casting process. The in-plane thermal conductivity of PI composite films with 7 wt% BNNSs is up to 2.95 W/mK, which increased by 1,080% compared to the neat PI. In contrast, the out-of plane thermal conductivity of the composites is 0.44 W/mK, with an increase by only 76%. The high anisotropy of thermal conductivity was verified to be due to the high alignment of the BNNSs. The PI/BNNSs composite films are attractive for the thermal management applications in the field of next-generation electronic devices.

## Introduction

With the rapid development of electronics industry, there is an increasing demand for electrically insulating polymer-based materials with enhanced capability of heat dissipation^1–5^. Furthermore, low cost and light weight polymer-based materials for next-generation electronic device, power systems, and communication equipment are needed. Polymers such as polyimide (PI) has been widely used as an electronic packaging material due to good thermal and mechanical properties^6,7^. In particular, it possesses a low dielectric constant, low loss tangent, high thermal stability and high storage modulus^8^. However, PI exhibit a poor thermal conductivity in the order of 0.1 W/mK^9–11^, which cannot meet the requirement of fast heat conduction for the advanced electronic devices. The general strategy to improve the thermal transport performance is using thermally conductive fillers such as carbon materials^12–15^, metal or ceramic materials^16–19^ are added to the polymer matrix^20^.

However, the carbon and metal materials are highly electrically conductive and small additions of these fillers into polymers result in high electrical conductivity of the composites, which restricts the application. Meanwhile, hexagonal boron nitride (h-BN) is a typical ceramic filler^21^ has attracted much attention due to its excellent electrical insulation and high thermal conductivity^22^. As the low aspect ratio of h-BN filler, conventional PI composite achieve the thermal conductivity of 1–5 W/mK by utilizing large loading volume fraction h-BN filler (of up to ~50%). Large loading volume fraction in polymer composites leads to many problems such as mechanical property deterioration and processing difficulty as well as cost enhancement. In the contrast, large aspect ratio boron nitride nanosheets (BNNSs) exhibit many potential applications including ultraviolet light emitter, field emitters, and a superior substrate for graphene-based electrical devices^23–28^ and a superior thermal conductivity ranges from 1,700–2,000 W/mK^29^. Furthermore, BNNSs are an electrical insulation material with dielectric constant of 2–4^30^. Therefore, BNNSs can be an ideal thermal conductive filler for polymer composites.

Recently, many efforts have been used to prepare BNNSs including micromechanical cleavage^31^, ultrasonication^32^, and high energy electron beam irradiation^33^, chemical vapor deposition^34^, and liquid exfoliation^35^. However, BNNSs still suffer from a low cost, high yield, and facile exfoliation method. Micromechanical cleavage and electron beam irradiation technique are inefficient and unscalable. Furthermore, chemical vapor deposition method usually involved expensive templates and complicated fabrication processes, which appears to be tedious and expensive for large-scale production. Though the liquid-exfoliation method is popular method, still suffers from low product yield and sometimes the toxic reagents have to be employed^36,37^. The high yielding and high-quality of BNNSs by exfoliation of h-BN powder remains a tough challenge.

Herein, BNNSs were prepared by a molten hydroxide assisted liquid exfoliation from h-BN powder. BNNSs with an average thickness of 3 nm were obtained in a high product yield of 19%^38^. This method has several advantages, such as cheap precursors, high yields, and without the use of organic solvents, catalysts and vacuum systems. More importantly, we developed a PI composite film incorporated with BNNSs. As a result, the incorporation of low loading BNNSs into the PI matrix shows a significant enhancement of thermal conductivity, especially along the in-plane direction. The composites are promising for using as a heat dissipation material in next-generation electronic device.

## Materials and Methods

### Materials

The h-BN powders were purchased from ESK Ceramics GmbH & Co. (Germany) with lateral size of 7 µm. Sodium hydroxide and potassium hydroxide were purchased from Sinopharm Chemical Reagent Co., Ltd (China). Poly(amic acid) synthesized by pyromellitic dianhydride and 4,4-oxydianiline was obtained from Ningbo Cen Electrical Material Co., Ltd (China). All chemicals were of analytical reagent grade and used without further purification.

### Preparation of BNNSs

The molten alkali-assisted exfoliation of h-BN was following by two steps. Firstly, NaOH (2.84 g) and KOH (2.16 g) were finely ground, and then h-BN micropowder (1.0 g) was added. The mixture was further ground into a homogeneous form and transferred to a 100 mL Teflon-lined stainless steel autoclave. The sealed autoclave was heated and kept at 180 °C for 2 h. After cooling down to room temperature, the solid product was collected from the autoclave and dispersed into 300 mL deionized water. The dispersion was sonicated for 1 h using a tip sonicator (SJIA-650, Ningbo Yinzhou Sjia Co., China). Subsequently the sample was filtered, re-dispersed in deionized water, and centrifuged to remove hydroxides and other unreacted materials. After centrifugation, the supernatant containing the product was collected. It is noted that the precipitate can be used for the next cycle of liquid exfoliation in the same alkaline solution as above mentioned.

**Figure 1:**
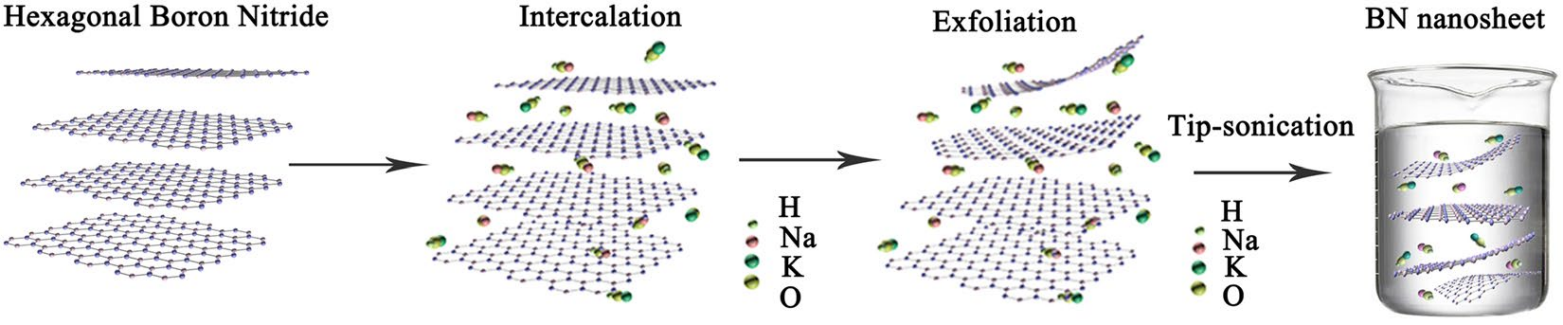
Schematic diagram of the exfoliation process.

To interpret the formation mechanism of BNNSs prepared by molten alkali-assisted exfoliation method, a schematic diagram is presented in Fig. 1. The early treatment of h-BN with molten alkali metal hydroxide was for the insertion of K^+^, Na^+^, and OH^−^ into the interlayer space of h-BN, driven by the high chemical potential. In particular, Na^+^ and K^+^ firstly absorbed on the surface of h-BN and then diffused into the space between adjacent BN lattices, leading to curling or crinkling of top BN layer^39^. After more ions entered the interlayer space, the curling up sheet peeled off from the parent counterpart owing to functionalized BN with hydroxides. This behavior enlarges the interlayer spacing near the edge. Then use molten hydroxide treated BN powder as precursor for liquid exfoliation and ultrasonication. The results indicate that the molten hydroxide treatment of BN powder could significantly improve the yield of BNNSs in the liquid exfoliation stage. The yield enhancement can be ascribed to the surface pretreatment, which effectively enlarges the BN sheets interlayer spacing and weaken the interaction between neighboring BN layers. Furthermore, the liquid exfoliation with dissolved hydroxides also induces the ion insertion, which further accelerates the exfoliation yield.

### Fabrication of PI/BNNSs composites

A solution method was adopted to prepare the PI/BNNSs composites. Firstly, BNNSs powders were dispersed in dimethylacetamide (1 mg/mL) by applying the ultrasonic wave for 10 min at room temperature. At the same time, poly(amic acid) was dissolved in dimethylacetamide at room temperature. Then, the BNNSs solution was added to a solution of poly(amic acid)/dimethylacetamide, and the resulting mixture was put into speedmixer to stirred for 4 min at 3200 rpm to obtain homogeneous mixture. Finally, the prepared mixture was casted on a clean glass substrate followed by thermally imidization in a vacuum oven at 80 °C for 2 h and 120, 150, 200, 250, 300 and 350 °C for 1 h, respectively.

### Characterization

The samples were examined with a Quanta FEG250 field emission scanning electron microscopy (FE-SEM, FEI, USA) at an acceleration voltage of 20 kV. Samples were broken and the fractured surface were coated with a thin layer of gold powder to avoid the accumulation of charge and improve the conductivity, and a transmission electron microscopy JEM-2100 (TEM, Jeol, Japan), respectively, with an acceleration voltage of 200 kV. The samples were dispersed in ethanol using ultrasonic mixing for 15 min and some pieces were collected on 200 mesh carbon coated copper grids. Atomic force microscope (AFM) measurement was conducted on a multimode scanning probe microscope from Digital Instruments with NanoscopeIa controller. X-ray photoelectron spectroscopy (XPS) was carried out with Kratos AXIS ULTR DLD spectrometer. Fourier transform infrared (FTIR) spectra were obtained using a Nicolet 6700 FTIR (Thermal scientific Inc. USA) between 400 and 4000 cm^−1^. Raman spectra were obtained by Raman spectrometer with laser wavelength of 532 nm (Renishaw plc, Wotton-under-Edge, UK). Thermal conductivities of the composites were determined with laser flash apparatus (LFA, Netzsch 447, Germany). The sample size for in-plane and out-of plane measurement was round with a diameter of 25.4 and 12.7 mm, respectively. The infrared (IR) photos were captured by IR camera (Fluke, Ti400, U.S.A.).

## Results and Discussion

### Characterization of BNNSs

**Figure 2:**
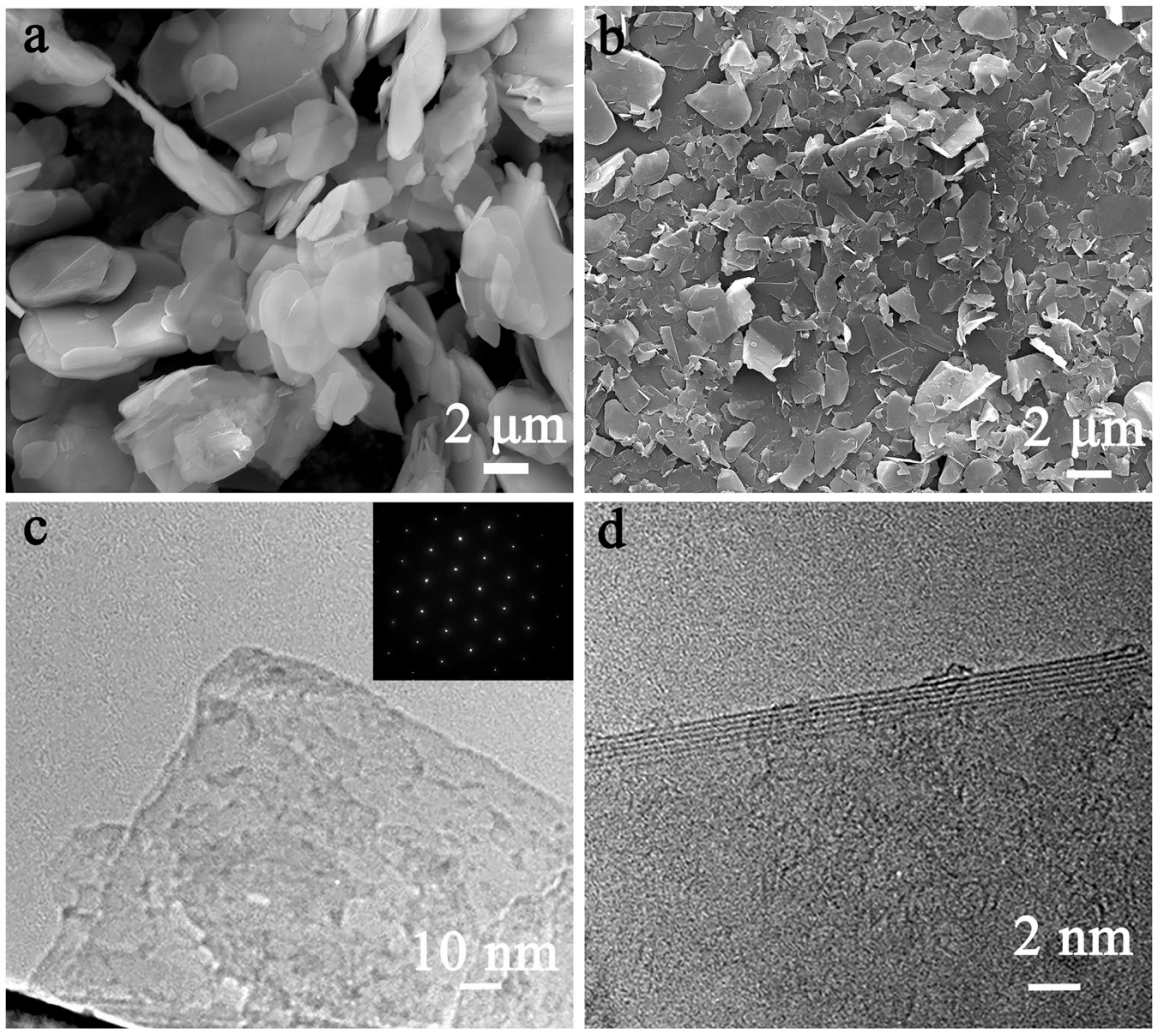
SEM images of (**a**) BN powder and (**b**) exfoliated BNNSs. (**c**) TEM images of BNNS and the corresponding electron diffraction pattern. (**d**) The high-resolution TEM image of BNNS.

The morphologies of the pristine BN powder and exfoliated BNNSs were studied using SEM. As shown in Fig. 2(a), the pristine BN powder shows a laminated structure with particle size of around 5–7 μm, and a plenty of BN sheets are stacked together. After exfoliation, much thinner and smaller BNNSs were observed in Fig. 2(b). Figure 2(c) is the TEM image of BNNS and the corresponding electron diffraction pattern displays a typical six-fold symmetry of BNNS due to the hexagonal crystal structure. Figure 2(d) shows the edge morphology of a single BNNS taken by TEM, which is similar to few-layer graphene^40^. The spacing between adjacent fringes and the number of layers was measured 8 layers, respectively.

**Figure 3:**
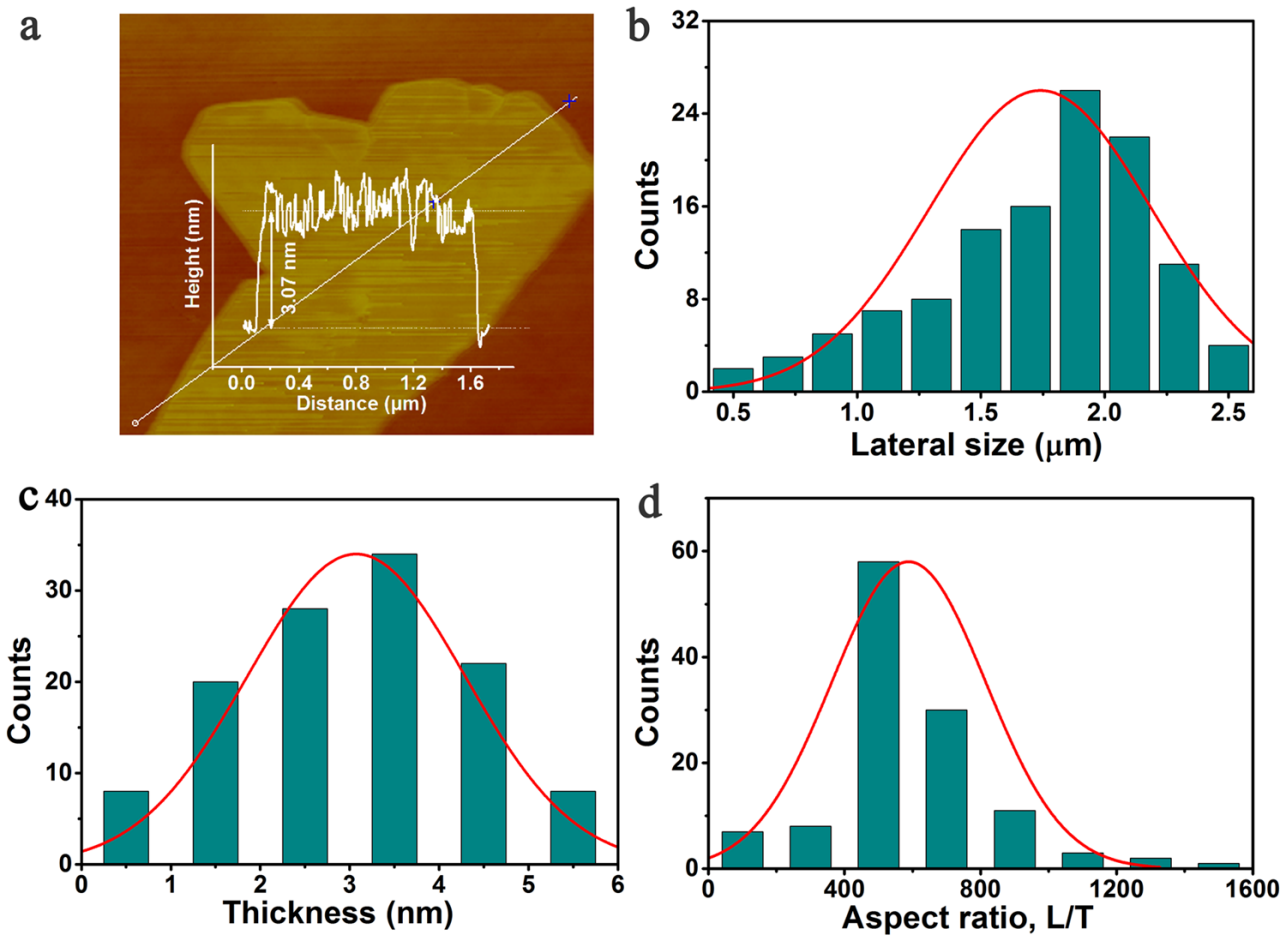
(**a**) AFM images of BNNS and height profiles, (**b**) histograms of measured values for nanosheets length, (**c**) histograms of measured values for nanosheets thickness, and (**d**) aspect ratio (length/thickness).

The thickness of the BNNS was approximately 3.07 nm, as shown in Fig. 3(a). These BNNSs can be assigned to a few-layered nanosheets, which is agreement with the TEM image in Fig. 2(d). The thickness (T) and length (L) of 120 pieces BN nanosheets were measured by analysing AFM images. The average lateral size of BNNSs is 1.8 μm as shown in Fig. 3(b). The thickness distribution of BNNSs was calculated and presented in Fig. 3(c). The average thickness of prepared BNNSs is 2.9 nm, which is in agreement with the TEM observation. The aspect ratio defined as nanosheets length over thickness, L/T, is an important factor in preparation of a freestanding heat spreader. Thus, the mean aspect ratio of BNNSs was 621 as shown in Fig. 3(d).

**Figure 4:**
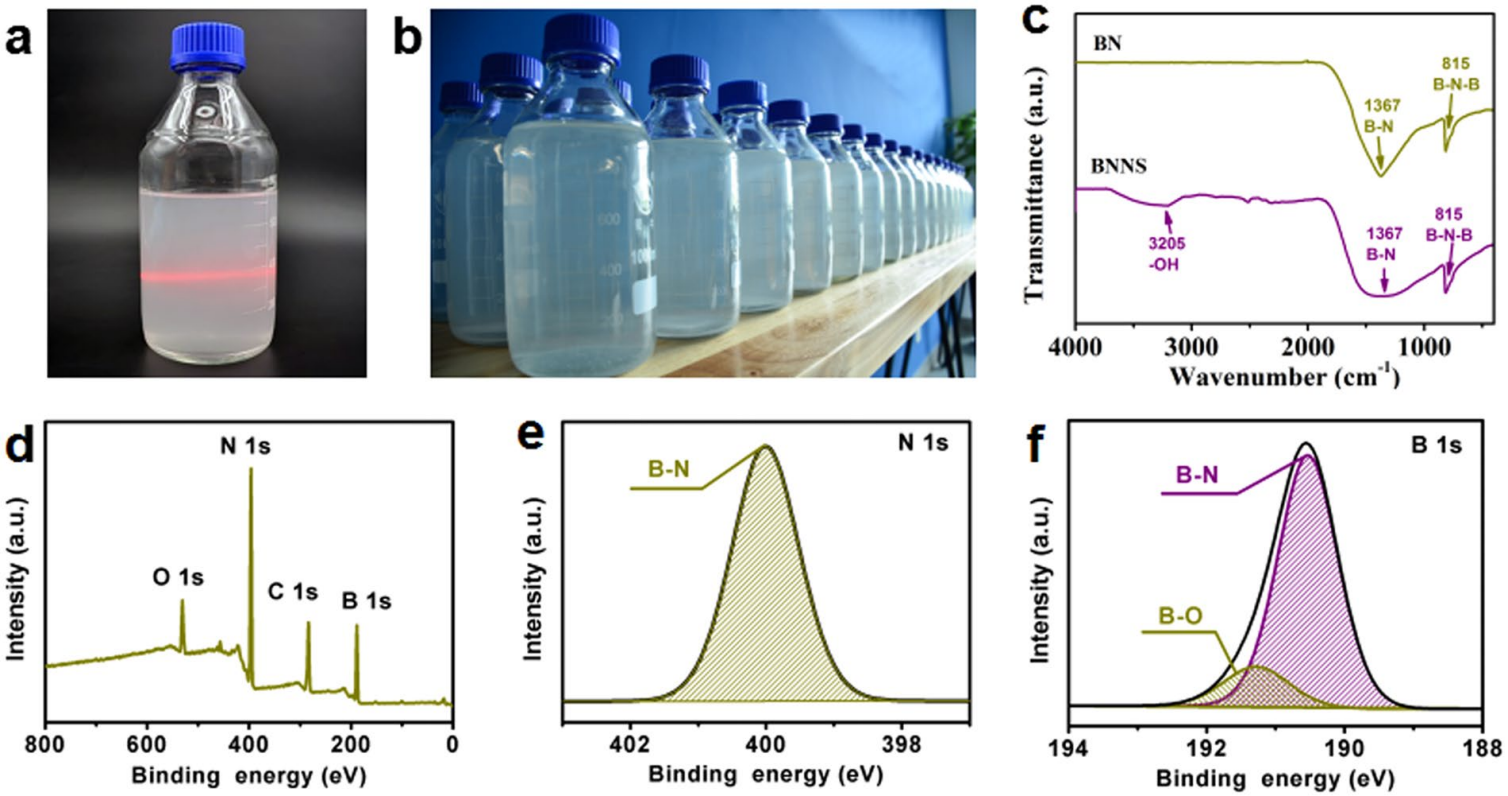
(**a**) Tyndall effect of suspensions of BNNSs in deionized water, (**b**) digital photo of produced BNNSs dispersions (1 L bottle, ~0.1 mg/mL), (**c**) FTIR spectra of pristine BN and exfoliated BNNSs, (**d**) XPS survey scan of BNNSs and corresponding (**e**) N 1s narrow XPS scans and (**f**) B 1s narrow XPS scans.

As shown in Fig. 4(a), the dispersion of exfoliated BNNSs with the irradiation of a laser beam from left to right shows a Tyndall effect because the lateral dimensions of the nanosheets are larger than the wavelength of the light and cause optical scattering effect. The Tyndall effect also confirms the colloidal nature and good stability of BNNSs dispersion in deionized water. Moreover, Fig. 4(b) shows significant potential to produce large-volume BNNSs dispersion. A low-cost and high-yield process based on molten alkali-assisted exfoliation is developed. Figure 4(c) shows the FTIR spectra of pristine BN and exfoliated BNNSs, respectively. Both FTIR spectra of BN and BNNSs exhibit strong absorption at 1367 cm^−1^, corresponding to B−N stretching (in-plane ring vibration, the E1u mode) and at 815 cm^−1^, which is attributed to B−N−B bending (out-of-plane vibration, A2u mode)^41,42^. However, the exfoliated BNNSs also exhibits a broad peak at 3205 cm^−1^, which is attributed to −OH vibration. This peak shows a significant decreasing after annealing process, indicating our proposed exfoliation method leads to hydroxyl functionalization of BNNSs. In order to investigate the surface composition and the functional groups, the XPS spectra of the BNNSs are shown in Fig. 4(d–f). The binding energy was calibrated with reference to the C 1s energy as 284.5 eV. In the XPS survey spectra of BNNSs, shown in Fig. 4(d), the peaks at 189, 396, and 531 eV correspond to B 1s of BNNSs, N 1s of BNNSs, and O 1s of hydroxyl functionalization of BNNSs and absorbed oxygen, respectively. Figure 4(e) indicates the N 1s core level spectrum can be fitted by one curve with binding energy of 399.9 eV, which is in good agreement with the previous reported values^43,44^. In addition, the B 1s core level spectrum in Fig. 4(f) show that the B−N peak centered at 190.5 eV and a B−O peak at 191.3 eV. The high-resolution B 1s scans indicate that N is only bound to B atoms, also suggesting that the oxidation is more favorable occur at boron sites^27,45,46^.

### The morphology of PI/BNNSs composites

**Figure 5:**
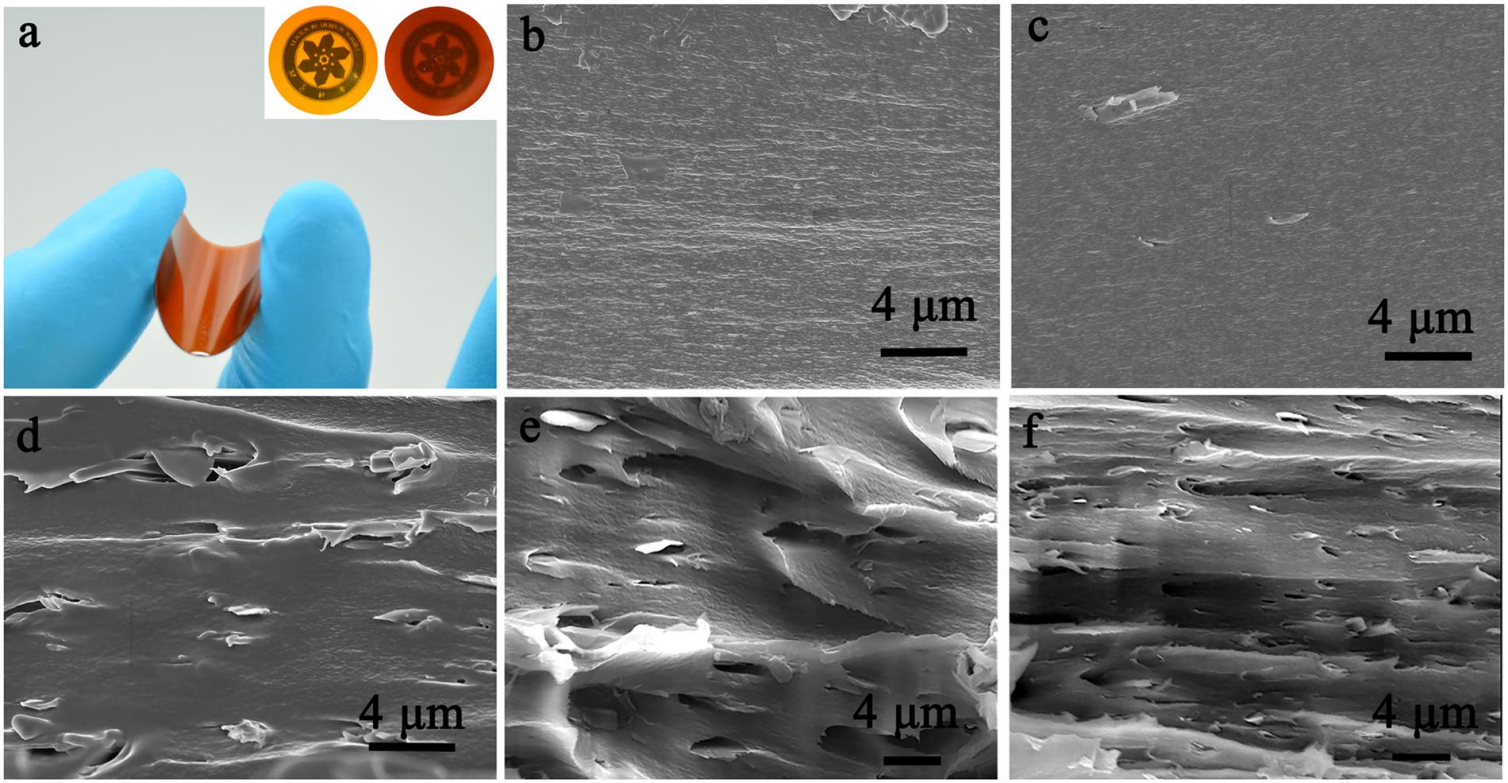
(**a**)The optical photograph of PI/BNNSs composites. Inset in (**a**) is the photographs of neat PI (left) and PI composites with 7 wt% BNNSs (right). SEM images of the fractured surface of (**b**) neat PI and PI/BNNSs composites with (**c**) 1 wt%, (**d**) 3 wt%, (**e**) 5 wt%, (**f**) 7 wt%.

The appearance of neat PI and PI composites are shown in Fig. 5(a). All as-fabricated PI/BNNSs composites film are prepared by a simply solution-casting procedure, resulting in large, free-standing papers, with about 100 µm thicknesses. Additionally, the paper was easily wrapped with hand, without noticeable damage to their structure, indicating their flexibility and durability. It is also found that the incorporation of BNNSs, even with high loading level of 7 wt%, does not lead to the visible reduction quickly, as the logo in the picture can be clearly seen in the inset of Fig. 5(a). Figure 5(b–f) shows the SEM photographs of the neat PI and PI composites containing 1, 3, 5 and 7 wt% of BNNSs, respectively. It can be seen that the fractured surface of neat PI is very smooth in Fig. 5(b). Obviously, the fractured surfaces of the PI composites are rougher compared with that of neat PI. Furthermore, BNNSs are homogenously dispersed and embedded in the matrix, as shown in Fig. 5(c–f), indicating good compatibility between the filler and matrix. With an increase in the BNNSs loading, one can clearly see more BNNSs with different shapes emerge at the fractured surface. Meanwhile, careful observation could find that the BNNSs had tendency to align in the PI matrix. This is because that the BNNSs possess unique two dimensional structure with large aspect ratio. The solution-casting method favored the parallel orientation of the BNNSs filler in the composites. Similar phenomenon can also be found in the previous works^47^.

### Thermal conductivity of PI/BNNSs composites

**Figure 6:**
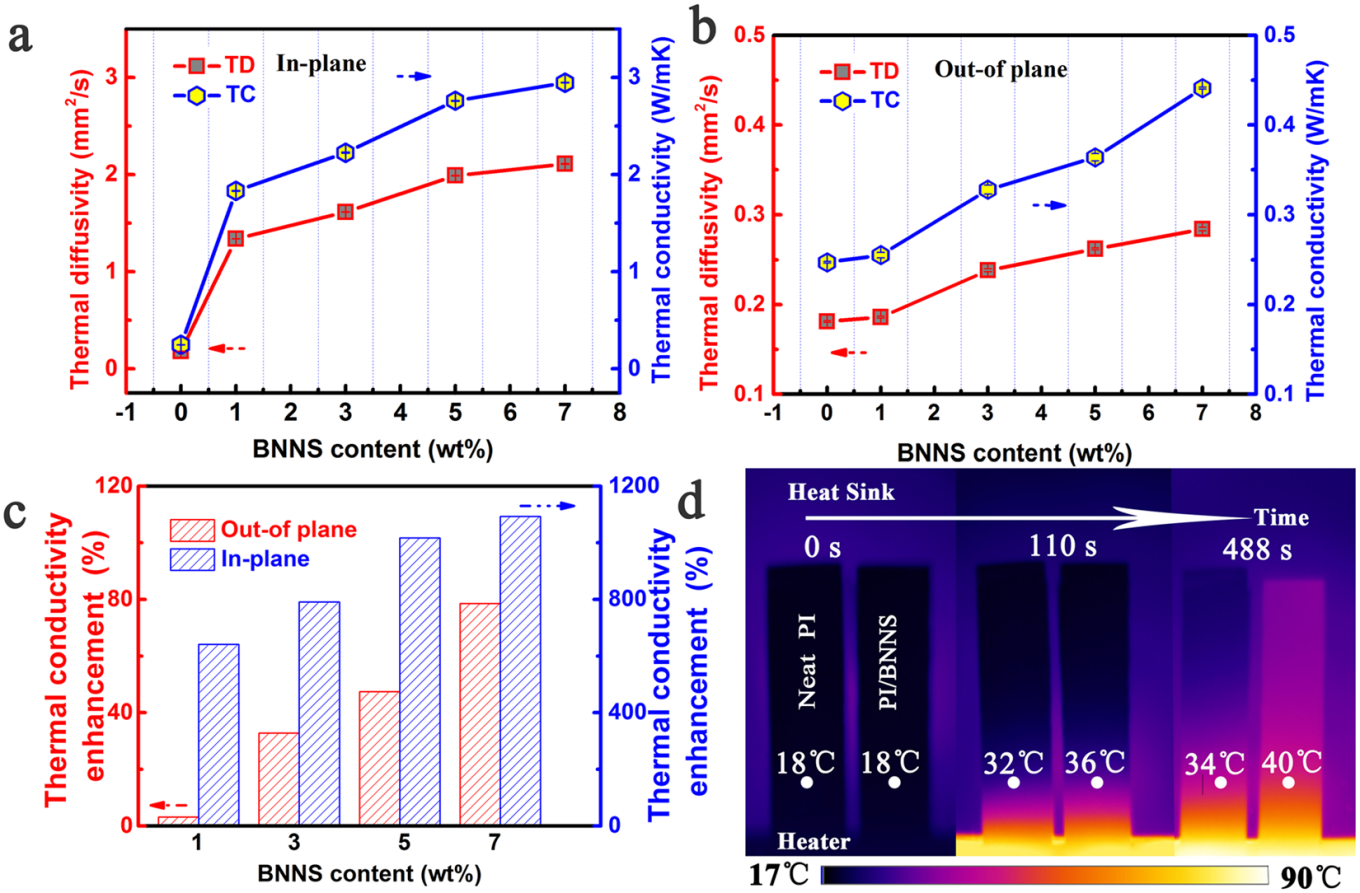
(**a**) Thermal diffusivity and thermal conductivity of neat PI and PI/BNNSs composite along in-plane direction. (**b**) Thermal diffusivity and thermal conductivity of neat PI and PI/BNNSs composite along out-of plane direction. (**c**) Thermal conductivity enhancement of PI/BNNSs composites compared to neat PI. (**d**) The temperature profile evolutions in time of neat PI and 7 wt% PI/BNNSs composite were captured using a calibrated infrared camera as a function of heating time.

Figure 6(a) and (b) show the thermal diffusivity and thermal conductivity of PI/BNN composite with the increasing BNNSs loading along the out-of plane and in-plane directions, respectively. It can be easily seen from Fig. 6(a) that the in-plane thermal conductivity of PI composites with 0, 1, 3, 5 and 7 wt% BNNSs fillers are 0.25, 1.83, 2.22, 2.76, and 2.95 W/mK, respectively. Meanwhile, Fig. 6(b) give the out-of plane thermal conductivity of PI composites are 0.25, 0.26, 0.33, 0.36, and 0.44 W/mK, respectively. The extraordinary increase along the in-plane direction, as the BNNSs loading in the PI which be attribute to the interconnected between BNNSs and the interfacial thermal resistance greatly reduced. However, the discontinuity of BNNSs leads to the high interfacial thermal resistance, which resulting lower increase for the out-of direction. As displayed in Fig. 6(c), a distinct contrast could be observed between the out-of plane and the in-plane thermal conductivity of PI composite films. We found that the out-of plane thermal conductivity of PI composite films with 7 wt% BNNSs fillers is 0.44 W/mK, which gives a slight improvement (76%), compared to that of neat PI (0.25 W/mK). Interestingly, PI composite films present the effective thermal conductivity enhancement along the in-plane direction, which increases up to 1080%. The value of 2.95 W/mK for the in-plane thermal conductivity of PI composite films can be achieved, with the same BNNSs fillers loading. The in-plane thermal conductivity of PI composite films is nearly seven (6.7) times to the out-of plane thermal conductivity, which indicate that PI/BNNSs composite films shows a highly anisotropic thermal property. The highly anisotropic thermal conduction of PI/BNNSs composites can be due to highly aligned BNNSs network throughout the PI matrix. A comparison of previous reported thermal conductivity values for other PI based composites or BN filled composites is obtained in Table S1. Moreover, the temperature profile evolution in time of two samples was recorded using a calibrated IR camera. Neat PI and 7 wt% PI/BNNS composite strips acted as the heat sink. The size of each strip was 20.0 (length) × 5.0 (width) × 0.2 mm (thickness). One end of the strip was connected to a heater. As shown in Fig. 6(d), IR images present the temperature increase of neat PI and PI/BNNSs composites from one side to another, respectively, as heating time goes. The temperature of two samples at the point with the same distance from the heater was compared. Before heating, the whole device stayed at 18 °C. When the heater worked, the PI/BNNS composite was heater than neat PI. When the heater reached 90 °C, the temperature of the PI/BNNSs composite film was 40 °C, which increased by 6 °C than 34 °C of neat PI film. This consequence give evidence of that BNNSs-reinforced PI films have better heat transportation performance in the real case, which is consistent with the higher thermal conductivity of PI/BNNSs composites along the in-plane direction.

**Figure 7:**
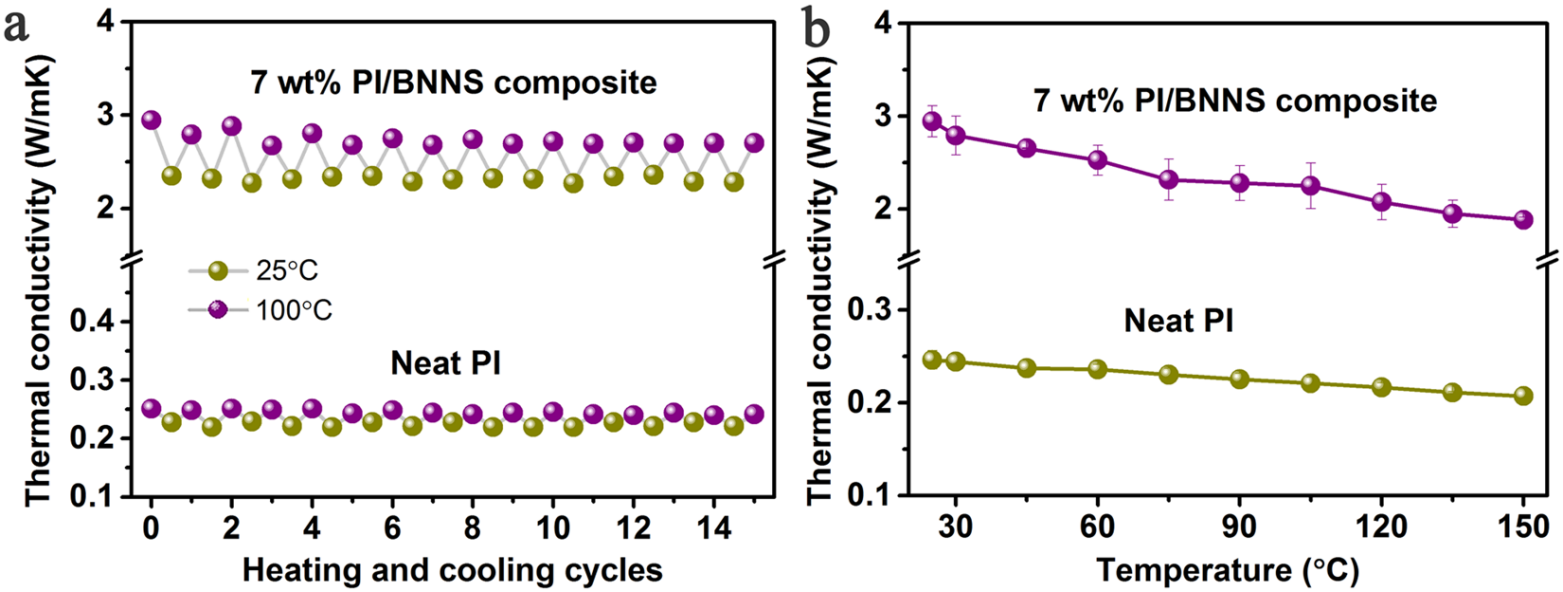
(**a**) Heating and cooling cycles of neat PI and PI/BNNSs composites. (**b**) Thermal conductivity of neat PI and PI/BNNSs composites as a function of temperature.

Figure 7(a) shows that heating and cooling cycles for neat PI and PI composites with 7 wt% BNNSs upon multiple heating and cooling cycles alternating between 25 and 100 °C. The thermal conductivity the neat PI and PI composites almost maintain the original thermal conductivity and exhibits a slight change within the fifteen cycles, suggesting stable capability of heat conduction in the range of this temperature. Such a small variation of thermal conductivity with temperature would be beneficial for the long-term device operation. Figure 7(b) shows the variation of in plane thermal conductivity of neat PI and PI composites with 7 wt% BNNSs as a function of temperature from 25 °C to 150 °C. The effective thermal conductivity for neat PI was found to be 0.25 W/mK at 25 °C and decreases with temperature over the temperature range investigated. The thermal conductivity of PI composites exhibits temperature dependences similar to the neat PI.

## Conclusions

In summary, BNNSs were prepared by a molten hydroxide assisted liquid exfoliation from h-BN powder. Highly thermally conductive PI/BNNSs composite films were produced by a simple solution-casting process. The in-plane thermal conductivity of PI composite films with 7 wt% BNNSs loading is up to 2.95 W/mK, which increased by 1,080% as compared to that of neat PI. In contrast, the out-of plane thermal conductivity of the composites is 0.44 W/mK, with an increase by only 76%. The thermal conductivity of PI/BNNSs composite shows highly anisotropy behavior according to the heat transfer direction. The PI/BNNSs composite films are very attractive for the thermal management (heat spreading) in electronics.

## Electronic supplementary material

Supplementary Information
