# Supplementary material for: Enhanced Thermal Conductivity of Polyimide Composites with Boron Nitride Nanosheets
Source: Sci Rep. 2018 Jan 24;8:1557. doi: 10.1038/s41598-018-19945-3 (PMC5784086; doi:10.1038/s41598-018-19945-3)
Supplement: Supplementary file 1 — Supplementary Information [file 41598_2018_19945_MOESM1_ESM.doc]

**SUPPORTING INFORMATION**

**Enhanced Thermal Conductivity of Polyimide Composites with Boron Nitride Nanosheets**

*Ting Wang^1#^, Mengjie Wang^1#^, Li Fu^2^, Zehui Duan^3^,Yapeng Cheng^1^, Xiao Hou^1^, Yuming Wu^1^, Shuangyi Li^1^, Liangchao Guo^1^,Ruiyang Kang^1^, Nan Jiang^*1^, Jinhong Yu^*1^*

*^1^Key Laboratory of Marine Materials and Related Technologies, Zhejiang Key Laboratory of Marine Materials and Protective Technologies, Ningbo Institute of Materials Technology and Engineering, Chinese Academy of Sciences, Ningbo 315201, China.*

*^2^College of Materials and Environmental Engineering, Hangzhou Dianzi University, Hangzhou, 310018, China.*

*^3^Chemical Engineering and Biotechnology, National Taipei University of Technology, Taipei, 10608, China.*

***^#^Ting Wang and Mengjie Wang contributed equally to this work.***

****Corresponding author, Electronic mail: jiangnan@nimte.ac.cn； yujinhong@nimte.ac.cn.***

Table S1.A comparison of various thermal conductive filler for polyimide composites.

| **Filler** | **TCE（%）** | **Thermal conductivity（W/mK）** | **Fraction (wt%)** | **Year and references** |
| --- | --- | --- | --- | --- |
| MgO | 12 | 0.155 | 1.5 | 2010^(1)^ |
| Micro-and nano-sized BN hybrids | 500 | 1.2 | 30 | 2010^(2)^ |
| Graphene | 27 | 0.108 | 2.5 | 2011^(3)^ |
| ZnO nanoparticles | 410 | 1.54 | 59 | 2011^(4)^ |
| S-MWNTs | 85 | 0.37 | 1.5 | 2012^(5)^ |
| Functionalized BN | 562 | 0.86 | 50 | 2012^(6)^ |
| Graphene oxide | 608 | 0.92 | 20 | 2013^(7)^ |
| Graphene | 294 | 1.002 | 11 | 2014^(8)^ |
| Functionalized-BN-50/ glycidyl methacrylate-grafted graphene-1 | 1500 | 2.11 | 51 | 2014^(9)^ |
| BN-coated multi-walled CNTs | 106 | 0.388 | 3 | 2014*(*^10)^ |
| Graphene/SiC hybrids | 989 | 2.63 | 11 | 2015*(*^11)^ |
| 1D SiC nanowires grown on 2D graphene sheets | 138 | 0.577 | 7 | 2015*(*^12)^ |
| γ-MPS-modified h-BN particles | 460 | 0.748 | 40 | 2016*(*^13)^ |
| Reduced graphene oxide | 52.9 | 0.26 | 2 | 2016 *(*^14)^ |
| Micrometer BN | 300 | 0.696 | 30 | 2017*(*^15)^ |
| BNNS | 1080  76 | 2.95 (in-plane)  0.44 (out-of plane) | 7 | This work |

**References**

1. K. Murakami, K. Yamada, K. Deguchi, T. Shimizu, S. Ando, Preparation of Soluble Polyimide/MgO Nanohybrid Films by In situ Hybridization Method and Evaluation of Their Thermal Conductivity. *Journal of Photopolymer Science and Technology* **23**, 501-506 (2010).

2. T.-L. Li, S. L.-C. Hsu, Enhanced thermal conductivity of polyimide films via a hybrid of micro-and nano-sized boron nitride. *The Journal of Physical Chemistry B* **114**, 6825-6829 (2010).

3. M. Koo *et al.*, Thermo-dependent characteristics of polyimide–graphene composites. *Colloid and Polymer Science* **289**, 1503-1509 (2011).

4. D. Yorifuji, S. Ando, Enhanced thermal conductivity over percolation threshold in polyimide blend films containing ZnO nano-pyramidal particles: advantage of vertical double percolation structure. *Journal of Materials Chemistry* **21**, 4402-4407 (2011).

5. I. Tseng, H. C. Lin, M. H. Tsai, D. S. Chen, Thermal conductivity and morphology of silver‐filled multiwalled carbon nanotubes/polyimide nanocomposite films. *Journal of Applied Polymer Science* **126**, 182-187 (2012).

6. M.-H. Tsai, I.-H. Tseng, J.-C. Chiang, J.-J. Li, Flexible polyimide films hybrid with functionalized boron nitride and graphene oxide simultaneously to improve thermal conduction and dimensional stability. *ACS applied materials & interfaces* **6**, 8639-8645 (2014).

7. I. Tseng, J. C. Chang, S. L. Huang, M. H. Tsai, Enhanced thermal conductivity and dimensional stability of flexible polyimide nanocomposite film by addition of functionalized graphene oxide. *Polymer International* **62**, 827-835 (2013).

8. W. Dai *et al.*, Enhanced thermal and mechanical properties of polyimide/graphene composites. *Macromolecular Research* **22**, 983-989 (2014).

9. M. H. Tsai, I. H. Tseng, J. C. Chiang, J. J. Li, Flexible Polyimide Films Hybrid with Functionalized Boron Nitride and Graphene Oxide Simultaneously To Improve Thermal Conduction and Dimensional Stability. *ACS Appl. Mater. Interfaces* **6**, 8639-8645 (2014).

10. W. Yan *et al.*, Polyimide nanocomposites with boron nitride-coated multi-walled carbon nanotubes for enhanced thermal conductivity and electrical insulation. *J. Mater. Chem. A* **2**, 20958-20965 (2014).

11. W. Dai *et al.*, Enhanced thermal conductivity for polyimide composites with a three-dimensional silicon carbide nanowire@ graphene sheets filler. *Journal of Materials Chemistry A* **3**, 4884-4891 (2015).

12. W. Dai *et al.*, Enhanced thermal conductivity and retained electrical insulation for polyimide composites with SiC nanowires grown on graphene hybrid fillers. *Composites Part A: Applied Science and Manufacturing* **76**, 73-81 (2015).

13. N. Yang *et al.*, Preparation and properties of thermally conductive polyimide/boron nitride composites. *Rsc Advances* **6**, 18279-18287 (2016).

14. L. Xu, G. Chen, W. Wang, L. Li, X. Fang, A facile assembly of polyimide/graphene core–shell structured nanocomposites with both high electrical and thermal conductivities. *Composites Part A: Applied Science and Manufacturing* **84**, 472-481 (2016).

15. J. W. Gu *et al.*, Dielectric thermally conductive boron nitride/polyimide composites with outstanding thermal stabilities via in-situ polymerization-electrospinning-hot press method. *Compos. Pt. A-Appl. Sci. Manuf.* **94**, 209-216 (2017).
